# Preprocedural Anxiety in Adults With Congenital Heart Disease: The PANIC Study

**DOI:** 10.1016/j.jacadv.2023.100589

**Published:** 2023-09-01

**Authors:** Stephen C. Cook, Arwa Saidi, Harsimran S. Singh, Ryan D. Madder, Scott B. Cohen, Stacie Van Oosterhout, Bennet P. Samuel, Michael T.M. Finn

**Affiliations:** a Indiana University Health Adult Congenital Heart Disease Program, Indiana University School of Medicine, Indianapolis, Indiana, USA; b Congenital Heart Center, University of Florida Health, Gainesville, Florida, USA; c Adult Congenital Heart Disease Program, Weill Cornell Medical Center, New York, New York, USA; d Division of Cardiology, Corewell Health, Grand Rapids, Michigan, USA; e Adult Congenital Heart Disease Program, Herma Heart Institute, Milwaukee Hospital-Children’s Wisconsin, Milwaukee, Wisconsin, USA; f Division of Cardiology, Corewell Health Office of Research and Education, Grand Rapids, Michigan, USA; g Karl and Patricia Betz Congenital Heart Center, Corewell Health Helen DeVos Children’s Hospital, Grand Rapids, Michigan, USA; h Independent Psychology Practice, Ann Arbor, Michigan, USA

**Keywords:** adult congenital heart disease, anxiety, elective catheterization, mental health, patient education

## Abstract

**Background:**

Preprocedural anxiety may have detrimental effects both cognitively and physiologically.

**Objectives:**

The objective of this study was to determine the association between state (situational) and trait (persistent in everyday life) anxiety and differences between the adult congenital heart disease (ACHD) and acquired heart disease populations.

**Methods:**

The State-Trait Anxiety Inventory and financial stress scale were administered to adults with acquired and CHD at 4 tertiary referral centers in the United States prior to cardiac catheterization. Student’s *t*-test and least absolute shrinkage and selection operator regression analyses were used to assess differences in anxiety between groups and identify the optimal model of predictors of anxiety.

**Results:**

Of the 291 patients enrolled, those with CHD (n = 91) were younger (age 41.3 ± 16.3 years vs 64.7 ± 11.3 years, *P* < 0.001), underwent more cardiac surgeries (*P* < 0.001), and had higher levels of trait anxiety (t[171] = 2.62, *P* = 0.001, d = 0.33). There was no difference in state anxiety between groups (t[158.65] = 1.37, *P* = 0.17, d = 0.18). State anxiety was singularly associated with trait anxiety. Trait anxiety was negatively associated with age and positively associated with state anxiety and financial stress. Patients with CHD of great complexity were more trait (*F*[2,88] = 4.21, *P* = 0.02) and state anxious (*F*[2,87] = 4.59, *P* = 0.01), though with relatively small effect size.

**Conclusions:**

Trait anxiety levels are higher in the ACHD population and directly associated with state anxiety. Specialists caring for ACHD patients should not only recognize the frequency of trait anxiety but also high-risk subgroups that may benefit from psychological or social interventions to reduce preprocedural anxiety.

Despite improvements in life expectancy for adults with congenital heart disease (ACHD), those surviving well into adulthood may be prone to experience psychosocial problems due to their lifelong medical condition. In North America, standardized psychiatric interviews with ACHD patients revealed 27% met criteria for a depressive episode and 9% met criteria for generalized anxiety disorder.^1^ Interestingly, these patients were felt to be ‘*well-adjusted*’ by their ACHD providers suggesting psychological distress is often under-recognized. Psychosocial challenges occur throughout the lifetime of the ACHD patient and include difficult transition to adulthood, heart-focused anxiety,^2^ negative thoughts associated with anxiety,^3^ and treatment-related worries. These may create challenges with regard to decision-making and preparation for outpatient procedures (eg, cardiac catheterization, radiofrequency, ablation, etc).

Preprocedural anxiety is present in up to 80% of patients.^4^ Physiologically, anxious patients may require more sedation because of a heightened perception of pain leading to lengthened recovery times. The cognitive effects of anxiety may also render patients unable to fully appreciate the improvements that have been achieved from transcatheter or surgical interventions.^5^ Previous studies evaluating preprocedural anxiety in patients undergoing angiography or percutaneous coronary intervention, demonstrated anxiety was highest immediately preprocedure, among female patients <65 years of age, in patients with lower levels of education and with acute percutaneous coronary intervention.^6^ Additional aspects contributing to anxiety at the procedure have included, fears of coronary artery bypass graft, uncertainty about the illness, pain, unfavorable clinical findings, lying flat in bed, and death.^7^

Preprocedural anxiety in ACHD patients remains poorly understood. A better understanding of preprocedural anxiety in ACHD patients may help guide the development of resources or strategies to lessen preprocedural anxiety. The purpose of this study was to determine the association between state (situational) and trait (persistent in everyday life) anxiety, clinical characteristics associated with higher anxiety levels, and differences between ACHD and acquired heart disease populations prior to cardiac catheterization.

## Methods

After Institutional Review Board approval had been obtained at all sites, patients ≥18 years of age were consented and prospectively enrolled prior to undergoing cardiac catheterization at 4 tertiary ACHD centers in the United States. A comparison group of adults without CHD undergoing coronary angiography or percutaneous intervention were also consented and prospectively enrolled prior to undergoing cardiac catheterization at a Midwest quaternary care center in the United States.

Baseline patient demographics including age, sex, race/ethnicity, body mass index, number of prior cardiac surgical interventions, number of prior cardiac catheterizations, level of education, and financial income were obtained on all patients. CHD diagnosis and complexity (simple, moderate, or great complexity) were also collected for ACHD patients.

Preprocedural anxiety was measured using the State-Trait Anxiety Inventory (STAI).^8^ The STAI evaluates 2 components of anxiety including: “state” anxiety (STAI-S) or anxiety occurring at a particular moment in time and an individual’s baseline level of anxiety, or “trait” anxiety (STAI-T). Overall, a total STAI score of 20 to 37 is consistent with no/low anxiety, a score of 38 to 44 represents moderate anxiety, and a score >45 represents high anxiety. Patients also completed a brief financial, chronic stress scale^9^ comprised of 3 questions (5-point Likert response scale) as the current literature continues to describe disparities in health in patients with limited socioeconomic resources.^10^

### Statistical analysis

Both *t*-tests and chi-square analyses were utilized to understand the degree of differences between the ACHD and acquired heart disease populations. More specifically, the goal was to evaluate the target anxiety variables of state and trait anxiety and how they varied across the 2 groups as well as the impact of CHD complexity on trait anxiety within the ACHD group.

#### Exploring for predictors of anxiety

In order to best discover optimal models of predictors to explain the target anxiety variables, cross-validated least absolute shrinkage and selection operator (LASSO) regression was utilized.^11^ The LASSO procedure fits an optimal generalized linear model by finding a set of variables that remain in a regularization path across a range of lambda values while accounting for penalty to be applied to the predictors. A cross-validated approach to LASSO regression performs the LASSO procedure across nonoverlapping samples of the data set to discover an optimal lambda value, at which tuning provides the optimal model of predictors of the outcome variable. The optimal LASSO lambda value reflects the optimal balance of variance and bias. Lower lambda values are more permissive of predictors. Thus, we selected the largest lambda value within 1 standard error of cross-validated errors for the minimal lambda value, making our exploratory values more conservative and parsimonious.

## Results

Of the 291 patients enrolled in the study, 58% were male (n = 168) with a mean age of 57.36 ± 16.9 years. Adult patients with CHD (n = 91) were younger (age 41.3 ± 16.3 years vs 64.7 ± 11.3 years, *P* < 0.001), underwent more cardiac surgeries (*P* < 0.001), included more women (57%, n = 52), and represented a more racially diverse (*P* = 0.053) population compared to adults with acquired heart disease. Table 1 shows the clinical characteristics of the entire study population.

**Table 1:** Baseline Patient Characteristics

	Overall (N = 291)	Acquired Heart Disease (n = 200)	Congenital Heart Disease (n = 91)	*P* Value
Age (y)	57.36 ± 16.90	64.67 ± 11.27	41.30 ± 16.12	<0.001
Male (%)	168 (57.7)	129 (64.5)	39 (42.9)	0.001
BMI (kg/m^2^)	29.71 ± 7.24	30.73 ± 7.26	27.48 ± 6.72	<0.001
Height (m)	1.70 ± 0.10	1.72 ± 0.10	1.67 ± 0.11	0.001
Weight (kg)	86.71 ± 23.12	90.89 ± 22.70	77.53 ± 21.43	<0.001
Non white (%)	26 (8.9)	13 (6.5)	13 (14.3)	0.053
Any postsecondary education (%)	174 (60.6)	114 (57.9)	60 (66.7)	0.199
Unreported	4	3	1	
Annual income ($50K or more) (%)	114 (47.7)	73 (45.1)	41 (53.2)	0.296
Unreported	52	38	14	
Number of prior cardiac surgeries	0.74 (1.14)	0.39 (0.76)	1.49 (1.45)	<0.001
Number of prior cardiac catheterizations	3.66 (7.22)	4.07 (9.07)	2.77 (3.01)	0.183
Financial stress	0.32 (0.25)	0.31 (0.25)	0.34 (0.26)	0.359
Unreported	34	20	14	
State anxiety	36.95 (11.23)	36.31 (10.82)	38.34 (12.04)	0.154
Trait anxiety	34.88 (9.75)	33.88 (9.59)	37.10 (9.79)	0.009

Values are mean ± SD or n (%).

**Figure 1:**
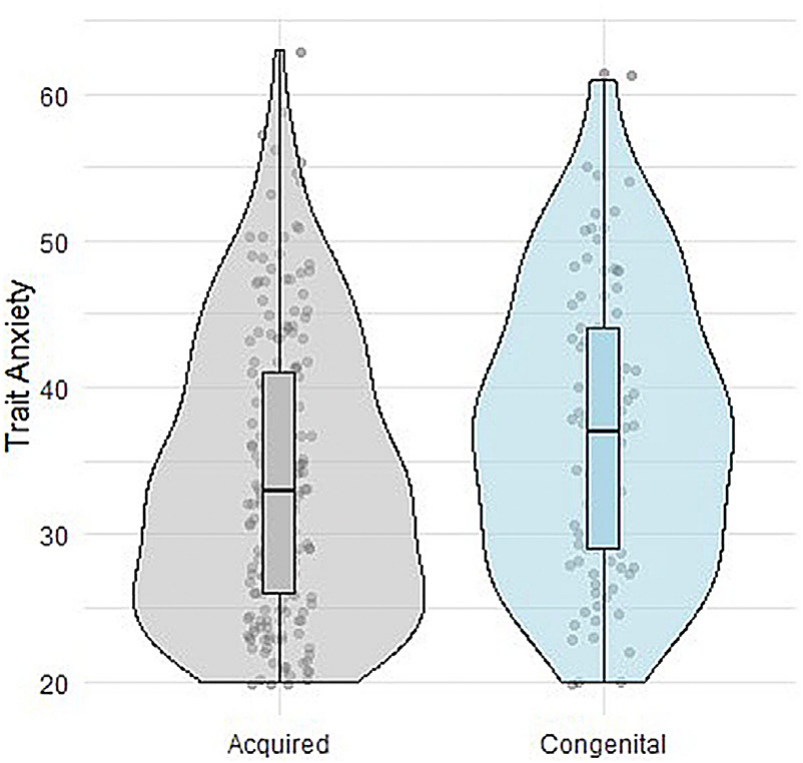
**Differences in Trait Anxiety** Violin plots demonstrating the differences between congenital and acquired heart disease groups on trait anxiety (t[171] = 2.62, *P* = 0.001, d = 0.3).

Compared to adults with acquired heart disease, ACHD patients had higher levels of trait anxiety (t[171] = 2.62, *P* = 0.001, d = 0.33) as shown in Figure 1. There was no difference in state anxiety between groups (t[158.65] = 1.37, *P* = 0.17, d = 0.18). Across groups, state anxiety was singularly associated with trait anxiety. Trait anxiety was negatively associated with age and positively associated with financial stress and state anxiety. ACHD patients with CHD of great complexity were more trait anxious compared to those with simple or moderate CHD complexity (*F*[2,88] = 4.21, *P* = 0.02) and, after removing one extreme outlier of simple complexity, also more state anxious (*F[*2,87] = 4.59, *P* = 0.01).

### Imputing data

In preparation for the exploratory models, which cannot be performed with partial cases, a small proportion of missing values (5.6% of the data set) were imputed so all patients had complete data on selected variables. Missing data occurred across the 3 individual items of the financial stress scale and 3 aspects of demographic data including income, current employment, and education level, (dichotomously coded). An important assumption to missing data imputation is that data are missing completely at random.^12^ We performed Little’s test of missing completely at random and found no evidence to suggest any discernible patterns in missingness, X2(59) = 67.5, *P* = 0.21. It appeared that missing data occurred randomly, perhaps due to the regular course of real-world implementation of a clinical study. We therefore proceeded with multivariate imputation by chained equations, a standard procedure for reliable imputation of data.^13^

### Exploring for anxiety predictors

Patients who were younger, had more financial stress, and had less income were observed to be more trait anxious (Table 2). In the exploratory model, having CHD vs acquired heart disease was no longer independently predictive of trait anxiety (lambda = 1.62). This indicated while patients with CHD were more likely to be trait anxious, upon further exploration their anxiety appeared more readily explained by younger age and financial stress.

**Table 2:** Exploratory Models of Trait Anxiety

Predictors	Overall			Congenital Heart Disease Only		
	Estimates	95% CI	*P* Value	Estimates	95% CI	*P* Value
(Intercept)	21.37	16.20-26.53	<0.001	20.48	12.43-28.52	<0.001
State anxiety	0.43	0.35-0.50	<0.001	0.36	0.22-0.49	<0.001
Age	−0.11	−0.16 to −0.06	<0.001	−0.13	−0.23 to −0.03	0.011
Financial stress	2.47	1.37-3.56	<0.001	3.01	1.22-4.79	0.001
Income [1]	−2.35	−4.10 to −0.59	0.009			
CHD complexity [moderate]				0.70	−3.39 to 4.80	0.734
CHD complexity [great]				3.84	−0.54 to 8.23	0.085
Observations	291			91		
R^2^/R^2^ adjusted	0.434/0.426			0.468/0.437		

Congenital heart disease (CHD) complexity variables are dummy-coded in comparison to simple complexity.

### State anxiety

State anxiety was also explored using the same cross-validated LASSO regression procedure and discovered only trait anxiety as a predictor (lambda = 3.27). Therefore, it appeared that anxiety on the day of catheterization was better explained by the patient’s more general trait anxiety, B = 0.64, SE = 0.06, *t*(289) = 11.46, *P* < 0.001, rather than a separate process perhaps more immediate to the day of catheterization.

### ACHD sample population

Finally, an exploratory LASSO regression was performed on the sample of ACHD patients in predicting trait anxiety. The variable of CHD complexity was included in this analysis. The same core set of predictors, state anxiety, age, and financial stress were noted. CHD complexity was observed to be present in the model (lambda = 2.29) (Table 2). In the sample of CHD patients, severity of CHD was noted to be a potentially important variable for explaining trait anxiety, though with a small effect size (Eta^2^ = 0.05) and relatively less important than age (Eta^2^ = 0.12) and financial stress (Eta^2^ = 0.12).

## Discussion

This is the largest prospective cohort to investigate preprocedural anxiety in adults with CHD. ACHD patients undergoing cardiac catheterization were observed to demonstrate elevated levels of trait anxiety compared to adults with acquired heart disease. Predictors of anxiety in ACHD patients also included younger age, CHD complexity, and financial stress. Importantly, state anxiety was exclusively predicted by trait anxiety.

Anxiety and depression are common among ACHD patients. Based on the results of studies using structured clinical interviews, the gold standard for psychiatric disorder diagnosis, approximately 31% of adults with CHD met criteria for a mood disorder (eg, major depressive disorder), and 28% were diagnosed with an anxiety disorder (eg, panic disorder or generalized anxiety disorder).^14^ Frequently, mood disorders and/or anxiety disorders are under-reported, under-recognized, and untreated often resulting in psychological distress associated with poorer quality of life and as a result adverse cardiovascular outcomes.^15^

Adults with CHD are faced with a set of unique challenges throughout their lifetime. These include transition from a pediatric to adult congenital cardiologist, navigating specialized care, cardiac symptoms, hospitalizations, diagnostic procedures, and need for recurrent surgical and transcatheter-based interventions. Consequently, many ACHD patients develop heart-focused anxiety. Heart-focused anxiety may include worry, avoidance, and attention specific to heart-related activity. It is usually accompanied by fear of heart-related sensations and symptoms and is often triggered by perceived negative consequences associated with feared sensations.^16^ In a prior retrospective study of ACHD patients who underwent psychological assessment, 71% patients reported heart anxiety, 49% reported difficulty coping with a medical decision as well as other health-specific concerns including treatment decision-making, surgical preparation, and adjustment to implanted devices.^17^

The results of this study clearly demonstrate elevated levels of trait anxiety in ACHD patients compared to patients with acquired heart disease. Furthermore, trait anxiety was greatest in those patients with CHD of greatest complexity. The relationship between anxiety and clinical outcomes in ACHD remains unknown. Prior studies have demonstrated the direct effects of anxiety on cardiovascular physiology and increased risk of incident cardiovascular disease.^18^ There are several behavioral and physiologic mechanisms that may explain the underlying associations between anxiety disorders and subsequent cardiovascular disease. Therefore, maintaining behaviors such as a healthy diet and an active lifestyle, as well as medication adherence, that may reduce cardiovascular risks associated with anxiety while simultaneously relieving and/or reducing anxiety symptoms. Thus, it was interesting to note that our cohort reporting increased trait anxiety was on average overweight or obese. Although we did not explore the relationships between trait anxiety, impact on lifestyle, and obesity, the role of anxiety and cardiovascular outcomes warrants further investigation in ACHD patients. For patients with heart failure, attendance at cardiac rehabilitation programs is an important step to improving quality of life and reducing risk of future hospitalization.^19^ Importantly, anxiety can lead to autonomic dysfunction. The disruption in the body’s ability to maintain heart rate variability and blood pressure has been shown to be important with regard to both overall cardiovascular health and mortality risk. Patients with acquired heart disease and autonomic instability are at increased risk for all-cause mortality.^20^ Although research is limited in ACHD patients, a prospective study of 171 patients with moderate/severe symptoms of major depressive disorder showed significantly lower heart rate variability.^21^ Decreased heart rate variability was associated with an increased risk of heart failure, hospitalization, and mortality. Additional physiologic mechanisms that may explain the association between anxiety and cardiovascular disease include inflammation,^22^ endothelial dysfunction,^23^ and platelet aggregation.^24^ These mechanisms may also be present and ‘activated’ in the anxious ACHD patient placing patients at increased risk for future cardiovascular events.

In this study, we found exploratory predictors of anxiety in ACHD patients compared to those with acquired heart disease that included: younger age and financial stress. While future studies should further investigate these relationships, specialists who provide care for ACHD patients should be knowledgeable of the high-risk patient with trait anxiety who may benefit from a proactive approach (eg, preprocedural education, psychology/social work assessment) to minimize preprocedural anxiety. Such assessments should be particularly sensitive to stressors in the social context of patients, such as financial stress and substantial disruptions to normative social development. It is well-known that adults with acquired heart disease with formal anxiety disorders are at risk for poor cardiovascular health and progression of cardiovascular disease. Given the relationships between cardiac outcomes and anxiety are mediated by the same behavioral and physiologic mechanisms in the ACHD patient, and that ACHD patients have a higher prevalence of trait anxiety, the diagnosis and treatment of anxiety disorders in ACHD patients is critical. Therefore, earlier referral to mental health professionals may play an important role in the identification of mood and anxiety disorders in ACHD patients (Central Illustration) Given the lack of access to mental health care due to scarcity of services and social stigma of mental health as well as financial resources required to receive appropriate treatments, additional work is required in this field to understand these common barriers (eg, access, stigma, affordability) to care in ACHD patients with mental health challenges.

**Central Illustration:**
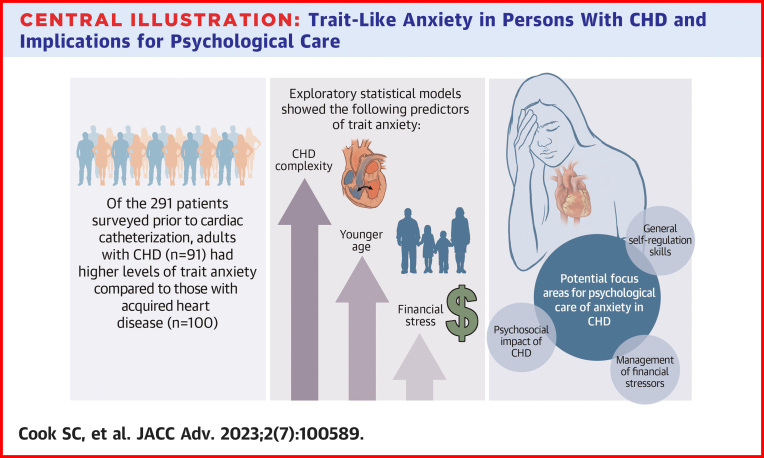
Trait-Like Anxiety in Persons With CHD and Implications for Psychological Care CHD = congenital heart disease.

Other factors outside the scope of this study that likely help explain elevated levels of trait anxiety in younger ACHD patients include the high prevalence of post-traumatic stress disorder (PTSD) in both adolescents and ACHD patients.^25^^,^^26^ Approximately 1 in 8 ACHD patients meet criteria for PTSD which is ∼50% higher compared to adults without CHD.^27^ In contrast to adults with acquired heart disease, it is not uncommon that children and adolescents with CHD require multiple, painful, or frightening procedures or hospitalizations throughout their lifetime. As a consequence, these experiences result in acute stress reactions or PTSD. From those studies, CHD patients diagnosed with PTSD were also found to have significantly elevated depressive symptoms and prior history of cardiac surgeries. This increased prevalence of anxiety, depression, and PTSD present throughout the lifetime of the CHD patient, should stress the importance to CHD providers for earlier identification (screening) and diagnosis of anxiety and other mood disorders in adolescents or preadolescents and especially before and following procedural interventions to improve the overall mental health of this susceptible population.

Providing preprocedural education may improve patient understanding about complex congenital procedures, decision-making competency, and ultimately reduce preprocedural anxiety in this vulnerable population. This study demonstrates trait anxiety is a good predictor of anxiety on the day of a procedure (R^2^ = 0.31) and may help to inform identifying patients at risk of severe state anxiety. A postprocedural visit with an advanced practice practitioner provides additional opportunities to provide patient education regarding procedural outcomes and/or interventions (eg, transcatheter pulmonary valve replacement, radiofrequency ablation for atrial flutter, etc) that the patient may not have had the opportunity to comprehend due to anxiety, sedation, or a combination of both. Future efforts for research in this field should include providing preprocedural education and early referral for mental health treatment to high-risk ACHD subgroups with anxiety and determine if these measures lead to faster recovery, decreased procedural anxiety, and improved health-related quality of life.

### Study limitations

This study has several limitations. Despite the prospective, multicenter approach to our study, the sample size of the total ACHD population is small in contrast to the number of adults with acquired heart disease. Still, our patients were obtained from 4 tertiary ACHD centers across the United States, suggesting trait anxiety is common among ACHD patients. The data were obtained through survey responses, which may be subject to response bias, recall bias, and selection bias. The Institutional Review Board at the center organizing the study required the survey questions to be optional resulting in missing data. However, the STAI is a well-validated tool that is commonly utilized to evaluate preprocedural anxiety and is designed to minimize bias from self-reported data.^28^

## Conclusions

Adults with CHD have high levels of trait anxiety compared to adults with acquired heart disease and experience preprocedural state anxiety in addition to their underlying trait anxiety. Upon exploration of contributing factors, it appeared that the highest levels of anxiety were experienced among younger patients, patients with CHD of great complexity, and those with greater financial stress. ACHD specialists should now be cognizant of the coexistence of anxiety disorders in ACHD patients and employ strategies to address coexisting mood or anxiety disorders that may alleviate preprocedural anxiety in this vulnerable population. A collaborative, patient-centered approach that incorporates mental health counseling as well as preprocedural and postprocedural education may offer the most successful opportunity to reduce anxiety, improve cardiovascular health, and improve health-related quality of life.

PERSPECTIVES **COMPETENCY IN MEDICAL KNOWLEDGE:** ACHD patients have higher trait anxiety levels than acquired heart disease patients undergoing similar procedures. Trait anxiety may have adverse effects on procedural outcomes.

**TRANSLATIONAL OUTLOOK 1:** These data strongly support efforts toward the early detection and treatment of trait-like anxiety, rather than focusing on anxiety that may only emerge on the day of a procedure. In order to work toward a high quality of life in ACHD patients, psychological care should focus on promotion of self-regulation of anxiety, the potentially disruptive nature of CHD to psychosocial development, and assistance in coping with financial stressors.

**TRANSLATIONAL OUTLOOK 2:** Additional research is now warranted to investigate efforts that include preprocedural education, mental health counseling as well as barriers to mental health care (stigma, access to care, affordability) to alleviate preprocedural anxiety leading to improved procedural outcomes and quality of life in ACHD patients.

## Funding support and author disclosures

The authors have reported that they have no relationships relevant to the contents of this paper to disclose.
